# Recent advances of cell therapies for AML/MDS: exploring the therapeutic potential of innate immune cells in AML/MDS

**DOI:** 10.1007/s12185-025-04113-x

**Published:** 2025-11-21

**Authors:** Yu-Hsuan Chang, Susumu Goyama

**Affiliations:** Division of Molecular Oncology, Department of Computational Biology and Medical Sciences, Graduate School of Frontier Sciences, The University of Tokyo, 4-6-1 Shirokanedai, Minato-ku, Tokyo 108-8639 Japan

**Keywords:** AML, MDS, Innate immunotherapy

## Abstract

Immunotherapy has revolutionized cancer treatments, yet checkpoint inhibitors and CAR-T cells have shown limited efficacy in acute myeloid leukemia (AML) and myelodysplastic syndromes (MDS). This limited efficacy is primarily due to the absence of lineage-specific antigens and the presence of suppressive microenvironments. Innate immunity offers a promising alternative, as it acts independently of neoantigens. The key players such as macrophages, dendritic cells, and natural killer (NK) cells not only eliminate malignant cells but also remodel the microenvironment to enhance antileukemic activity. This review highlights recent advances in understanding innate immune mechanisms in AML and MDS and explores therapeutic strategies designed to leverage these pathways, aiming to broaden the scope of immunotherapy.

## Introduction

Acute myeloid leukemia (AML) and myelodysplastic syndrome (MDS) are hematological malignancies originating from hematopoietic stem and progenitor cells (HSPCs) in bone marrow. AML is characterized by uncontrolled proliferation of leukemia blasts, while MDS is characterized by ineffective hematopoiesis and a potential risk of progression to AML. Although conventional treatments, such as chemotherapy and hematopoietic stem cell transplantation, have improved over the past few decades, approximately 50% of the adult AML cases ultimately relapse due to treatment failure [1]. Therefore, developing novel and more effective treatment strategies is an urgent priority.

In recent years, cancer immunotherapy has transformed oncology by harnessing the immune system to target malignancies. Research in cancer immunotherapy has predominantly focused on adaptive immune responses, particularly the role of T cells in tumor recognition and clearance. These studies have led to significant advancements, such as immune checkpoint blockade and Chimeric antigen receptor (CAR)-T cell therapies, which have demonstrated remarkable efficacy in various cancers, including lymphoid malignancies. However, despite these successes, immune checkpoint blockade and CAR-T cell therapies have shown limited efficacy against myeloid tumors. Several factors contribute to this resistance. First, AML and MDS typically exhibit a relatively low mutational burden, resulting in fewer neoantigens available for T cell recognition. Second, unlike B cell tumors with clear targets such as CD19, no similarly specific target antigens have been identified for myeloid malignancies. These challenges, combined with the immunosuppressive microenvironment characteristic of myeloid tumors, underscore the need for novel approaches to improve therapeutic outcomes.

Innate immunity recently has gained recognition as a key determinant of cancer control. Unlike adaptive immunity, it operates independently of neoantigens, making it particularly relevant in low-mutational burden tumors such as AML and MDS. Innate immune cells, including macrophages, dendritic cells, and natural killer (NK) cells play critical roles in recognizing and eliminating malignant cells, as well as modulating the tumor microenvironment to support antitumor activity. Harnessing these mechanisms has the potential to overcome some of the limitations of adaptive immune-based therapies.

In this review, we focus on the significance of innate immunity in the context of AML and MDS. We aim to provide a comprehensive overview of recent advancements in our understanding of innate immune mechanisms and their therapeutic potential. Furthermore, we discuss the latest developments in antileukemic therapies targeting innate immune pathways, offering insights into novel strategies to enhance cancer immunotherapy.

## The role of innate immune cells in AML/MDS

### NK cells

NK cells are innate lymphocytes with potent cytotoxic activity that play a critical role in immune surveillance. They detect and eliminate infected or abnormal cells that lack major histocompatibility complex (MHC) class I expression through the “missing-self” recognition mechanism. The number and cytotoxic activity of NK cells in patients with MDS and AML are significantly lower than those in healthy individuals [2, 3]. Functionally, NK cells from AML/MDS patients demonstrate multiple exhaustion phenotypes, including reduced activating receptors, elevated inhibiting receptors, impaired antibody-dependent cellular cytotoxicity (ADCC) activity and cytolytic function. Perforin, granzyme B, and interferon-γ (IFN-γ) secreted from NK cells are significantly weakened in AML/MDS patients [2, 4]. Furthermore, leukemia stem cells (LSCs) in AML do not express ligands that bind to the activating receptor NKG2D on NK cells, suggesting that AML stem cells can evade NK cell immune recognition [5]. These studies have established the importance of NK cells in AML immune surveillance and also partly explain why residual leukemia cells induce disease recurrence by evading NK cells after conventional treatments.

**Fig. 1:**
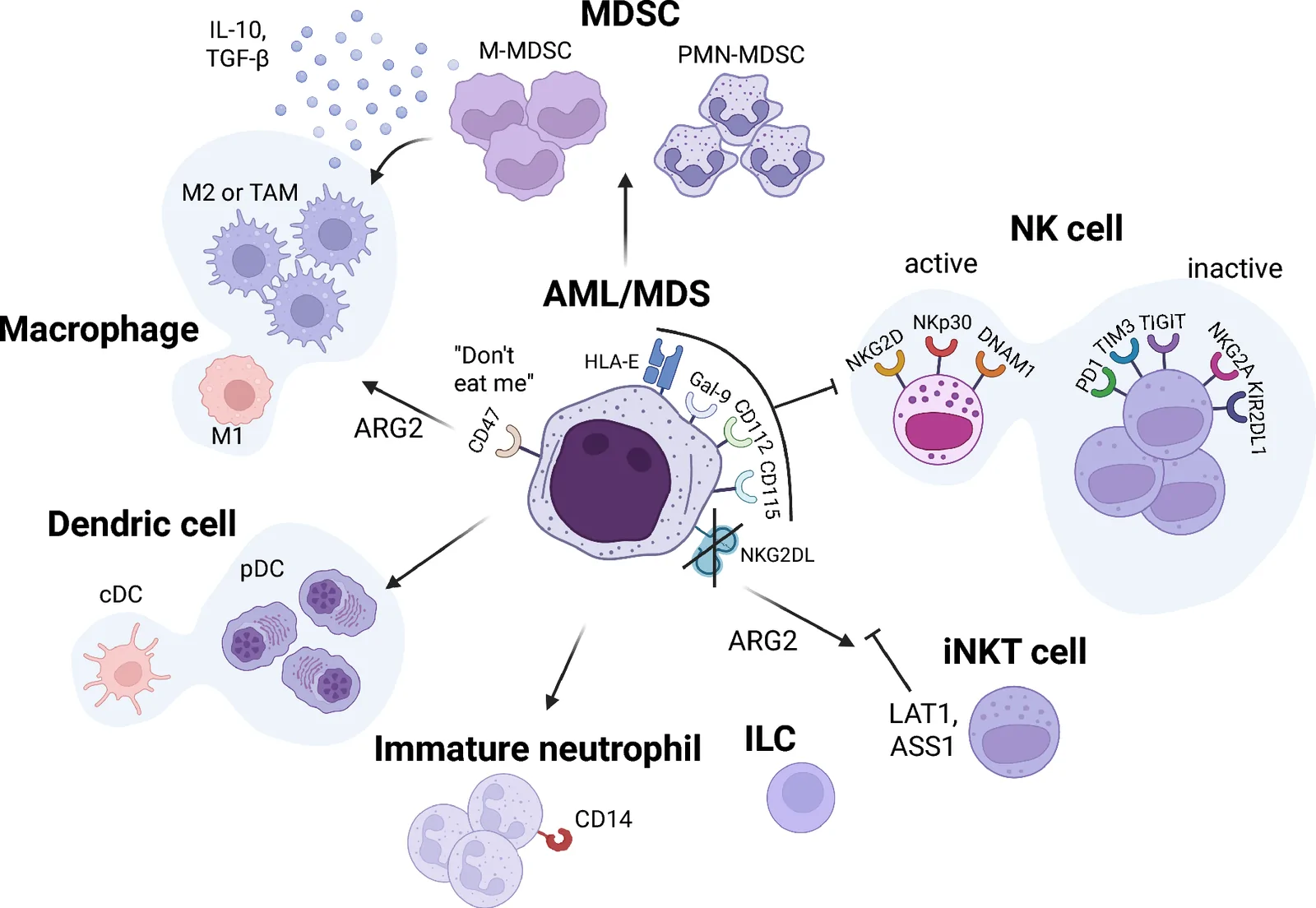
Innate immune dysfunction in the microenvironment of AML and MDS. The MDSCs, TAMs, M2-like macrophages, pDCs and inactive form of NK cells are increased in the patients with AML/MDS. MDSC: myeloid-derived suppressor cells, M-MDSC: monocytic MDSC, PMN-MDSC: granulocytic or polymorphonuclear MDSC, TAM: tissue-associated macrophage, cDC: conventional DC, pDC: plasmacytoid DC, ILC: innate lymphoid cell, iNKT: invariant natural killer T cell, ARG2: arginase-2, NKG2DL: NKG2D ligands

Over the past decade, there has been a deeper understanding of the molecular mechanisms underlying NK cell dysfunction in AML/MDS. Leukemia cells highly express inhibitory ligands, including CD137L, Galectin-9, CD112, and CD155, which bind to corresponding receptors on the surface of NK cells, thereby inhibiting NK cell cytolytic activity and IFN-γ production [6, 7]. In addition, several immune checkpoint inhibitory receptors on NK cells, such as programmed death receptor 1 (PD-1), TIGIT, and TIM-3, which are significantly higher from newly diagnosed AML patients compared to healthy individuals [8] (Fig. 1). These findings enhance our understanding of the immunosuppressive ability of leukemia and provide new insights into therapeutic strategies.

The antileukemic activity of NK cells was first demonstrated by Ruggeri et al., who observed that AML patients whose donor-derived NK cells were alloreactive in the graft-versus-leukemia (GVL) direction had significantly improved survival [9]. To enhance the antileukemic function of NK cells, compound treatment or gene-editing technology have been used for AML/MDS treatments combined with NK cells. The low dose of hypomethylating agents, such as azacitidine or decitabine, increases the expression of NKG2D- and DNAM1-activating ligands on AML cells and enhances the secretion of inflammatory cytokines by NK cells, thereby maximizing the cytotoxic effects of HSPC-derived NK cells [10]. Moreover, the p53 activator reduces leukemia cell growth in collaboration with NK cells in a mouse AML model [11, 12]. Furthermore, we recently found that *SETDB1* depletion in AML/MDS cells significantly upregulates the expression of NKG2D ligands while downregulating MHC-I expression, resulting in increased susceptibility to NK cell-mediated cytotoxicity [13]. The depletion of inhibitory receptor *KLRC1/NKG2A* on NK cells has been demonstrated to increase cytotoxicity against HLA-E expressed AML cells [14]. The inactivation of IL15-signaling negative regulator *CISH* in NK cells also extends the survival of HSPC-derived NK cells and exerts the preferential cytotoxicity in mice [15]. These studies indicate that deletion of inhibitory pathways within NK cells enables them to bypass the tumor immunosuppressive environment and enhance their antileukemic ability. Therefore, these findings provide therapeutic potential of combination treatments with adoptive NK cell transfer.

### Myeloid-derived suppressor cells and tumor-associated macrophages

Myeloid-derived suppressor cells (MDSCs) are a heterogeneous population of immature bone marrow-derived myeloid cells that expand under pathological conditions, including chronic inflammation, infection, and malignancy. MDSCs can be primarily divided into two distinct subsets: monocytic MDSCs (M-MDSCs) and granulocytic or polymorphonuclear (PMN-MDSCs) [16]. Additionally, in the classical binary model, macrophages are polarized into either a pro-inflammatory M1 phenotype or an immunosuppressive M2 phenotype, while contemporary perspectives recognize macrophage function in tumors as a continuum, not a strict dichotomy [17]. Early studies showed that tumor-associated macrophages (TAMs) are supposed to originate from monocytes or tissue-specific macrophages and predominantly exhibit M2-like characteristics, which correlate with tumor progression and poor prognosis [18]. MDSCs and TAMs are not merely two independent populations. Research indicates that the M-MDSC subset can differentiate into TAMs within the tumor microenvironment [19]. This important association suggests that MDSCs provide a continuous supply of TAMs within the tumor. Several research groups have reported an increased proportion of M2-like macrophages in the bone marrow of AML patients, while M1-like macrophages with antileukemic activity were relatively scarce [20]. This imbalance in macrophage subsets may contribute to an immunosuppressive niche that favors the survival of leukemia cells (Fig. 1).

TAMs and MDSCs are central mediators of tumor-induced immunosuppression and share several mechanisms. These mechanisms amplify tumor-induced immunosuppression, including the expression of arginase-1 (ARG1) and indoleamine 2,3-dioxygenase (IDO), which deplete the critical amino acids for T cell function, as well as the secretion of TGF-β and IL-10 that suppress effector T cells and promote the expansion of regulatory T cells (Treg) [21]. Additionally, inflammatory mediators secreted by stromal cells, such as S100A8/A9, and IL-6, drive suppressor cell differentiation and rewire metabolisms to stabilize the functions of TAMs and MDSCs [22]. Collectively, the immunosuppressive environment maintained by MDSCs, TAMs, and stromal cells further promotes leukemia cell survival and therapy resistance (Fig. 1).

### Dendritic cells

Dendritic cells (DCs) are specialized antigen-presenting cells that bridge innate and adaptive immunity. They are broadly classified into conventional DCs (cDCs, derived from myeloid progenitors) and plasmacytoid DCs (pDCs, derived from myeloid or lymphoid progenitors). cDCs are generally associated with robust T cell infiltration and favorable prognosis, while pDCs promote immune suppression and correlate with poor outcomes through IL-10 secretion and IDO-mediated Treg induction [23]. In AML/MDS, cDCs are markedly reduced in bone marrow and peripheral blood, limiting their capacity to cross-present and prime cytotoxic T lymphocytes [24] (Fig. 1). The deficiency in DC number and function contributes to impaired CD8⁺ T cell responses, facilitating immune evasion by leukemia cells [24]. This phenomenon provides the feasibility of therapeutics by dendritic cell vaccines for AML/MDS patients. The recent advances and clinical trials of DC-based therapies in AML are summarized in the referenced review [25].

### Neutrophils

Neutrophils, eosinophils, and mast cells are granular leukocytes traditionally associated with innate defense against pathogens, parasites, and allergens. Recent scRNA-seq analyses using neutrophil-reporter mice in the *Meis1-HoxA9* AML model revealed impaired neutrophil maturation and reduced frequency [26]. AML-associated neutrophils display an immature, CD14⁺ phenotype with monocytic and inflammatory features driven by NF-κB signaling [26]. Functionally, these CD14⁺ neutrophils suppressed T cell proliferation compared to neutrophils from healthy controls, indicating that leukemia-associated neutrophils contribute to the immunosuppressive marrow microenvironment [26] (Fig. 1).

### Innate lymphoid cells and iNKT cells

Innate lymphoid cells (ILCs) are a class of emerging lymphocytes that lack T cell or B cell receptors, primarily divided into three categories: ILC1 (express T-bet and produce IFN-γ), ILC2 (express GATA3 and produce IL-5 and IL-13), and ILC3 (express RORγt and secrete IL-17 and IL-33), and their functions are similar to those of their T cell counterparts (Th1, Th2, Th17). The analysis of bone marrow and peripheral blood from MDS patients revealed a near-complete loss of ILC2 and ILC3 subsets in the marrow compared to healthy controls, suggesting disruption of the ILC maturation continuum as a potential contributor to MDS immunopathogenesis [27]. In contrast, ILC1 frequencies remain largely preserved in both bone marrow and peripheral blood, showing no significant differences from healthy individuals [27]. A pathogenic role for ILC2s has previously been identified in acute promyelocytic leukemia (APL), showing that tumor-derived prostaglandin D2 (PGD2) activates ILC2s via the CRTH2 receptor, resulting in IL-13 secretion and subsequent activation of monocytic MDSCs, thereby establishing an immunosuppressive microenvironment [28]. Additionally, mesenchymal stem cells (MSCs) from AML patients produce PGD2 as well, which drives ILC2 proliferation and secretion of IL-5 and IL-13 [29]. Notably, IL-5 was found to expand Tregs and promote proliferation of both normal and malignant HSPCs, highlighting a stromal-driven PGD2-ILC2-Treg axis that supports leukemic progression [29]. These findings suggest that therapeutic targeting of PGD2-CRTH2 or IL-5 pathways may disrupt this circuit and inhibit leukemic proliferation.

Invariant natural killer T (iNKT) cells are a rare subset of T lymphocytes that co-express a semi-invariant TCR and NK receptors, recognizing lipid antigens via CD1d in a non-MHC-restricted manner [30]. They mediate antileukemic activity through direct cytotoxicity and cytokine-driven immune regulation that enhances dendritic cell maturation, CD8⁺ T cell priming, and NK cell recruitment [30]. Although leukemic niches rich in arginase-2 (ARG2) suppress conventional T cells, iNKT cells sustain function by upregulation of specific amino acid pathways, including large neutral amino acid transporter 1 (LAT1) and argininosuccinate synthase 1 (ASS1), maintaining proliferation and cytotoxicity under arginine deprivation [31] (Fig. 1). This metabolic resilience supports the therapeutic potential of iNKT cells against AML.

## Therapies against AML/MDS targeting innate immune cells

### NK cells

Several research on enhancing antileukemic activity of NK cells has been undergoing preclinical studies and clinical trials.

#### Checkpoint blockade strategies and cytokine-based approaches

**Fig. 2:**
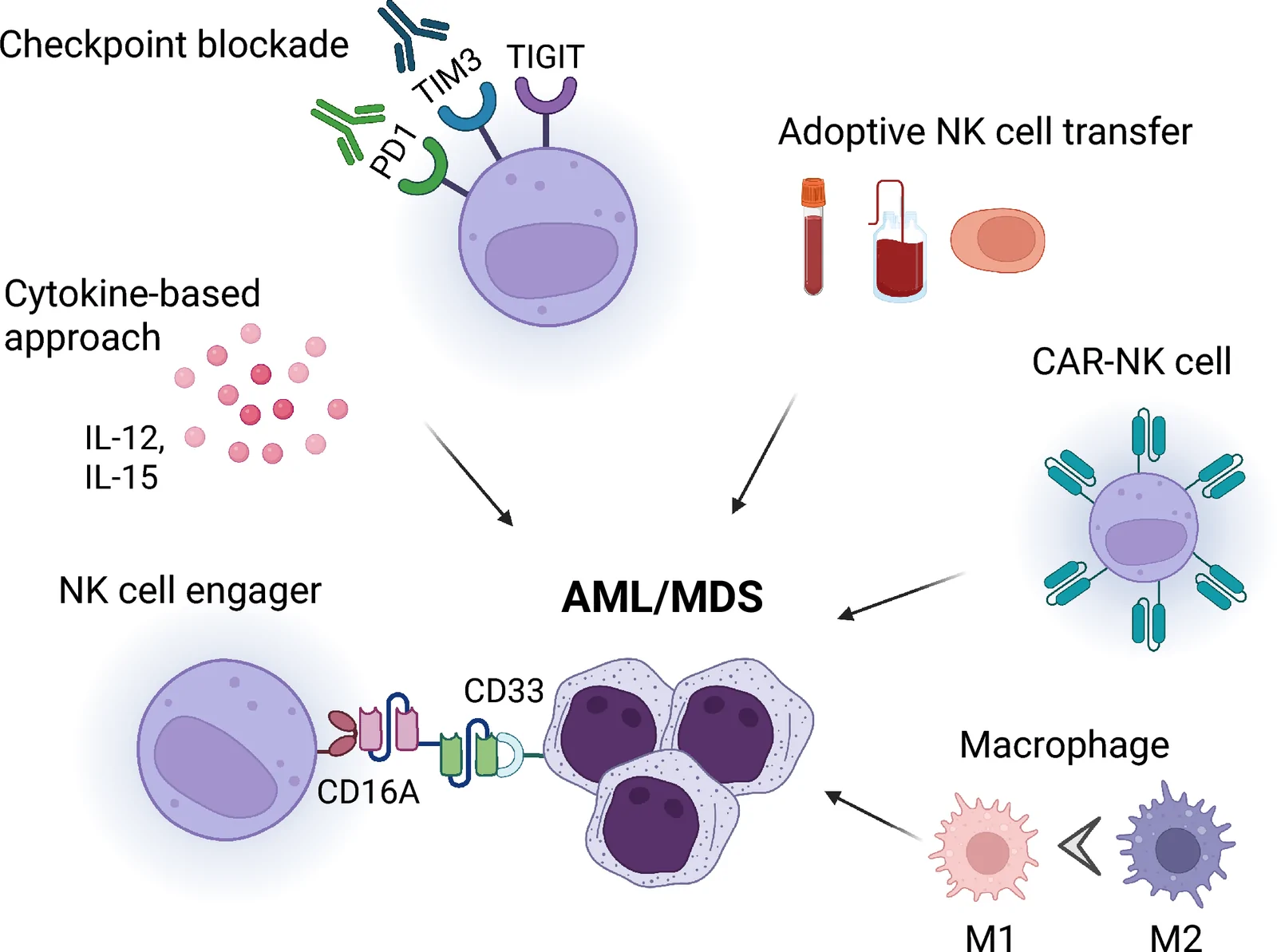
Harnessing innate immunity in the treatment of AML and MDS. Checkpoint blockades, cytokine-based approaches, NK cell engagers, adoptive NK cell transfer, and CAR-NK cell therapies are currently being evaluated in clinical trials. Approaches to reduce M2-like macrophages or repolarize M2 into M1 macrophages are also under investigation

Checkpoint blockade strategies, such as anti-PD-1, anti-CTLA4, and anti-TIM-3 antibodies, aim to restore NK cell activity [32]. Cytokine-based approaches, particularly IL-15 and IL-12, are also under investigation for enhancing NK cell proliferation and function [33, 34] (Fig. 2). Beyond NK cells, IL-12 can reprogram MDSCs, DCs, and macrophages toward antigen-presenting phenotypes and facilitate CD8⁺ T cell infiltration. IL-12 was also shown to enhance the cytotoxicity of peripheral blood mononuclear cells, thereby reshaping the tumor microenvironment in murine melanoma models [35, 36]. Further studies are needed to investigate the safety and effectiveness of using cytokines in clinical practice.

#### NK cell engagers

MDSCs and TAMs cooperatively suppress NK- and T-cell antileukemic activity. To overcome this, NK cell engagers have been developed. Bispecific T-cell engagers (BiTEs) connect CD3 on T cells with tumor antigens, inducing potent cytotoxicity but often causing cytokine release syndrome (CRS). Bispecific killer engagers (BiKEs) redirect cytotoxicity of NK cells by linking CD16a with tumor antigens, activating NK cell-mediated tumor killing while reducing CRS risk. Trispecific killer engagers (TriKEs) further embed IL-15 into the BiKE framework, enhancing NK cell survival and proliferation in vivo. For instance, CD33-directed BiKE/TriKEs offer dual benefits for the treatment of AML/MDS, as CD33 is expressed on both AML blasts and MDSCs [37, 38] (Fig. 2). CD33-directed BiKE/TriKEs not only promote NK-mediated leukemia cell killing but also deplete CD33^+^ MDSCs, thereby removing a key barrier to NK cell function [37, 38]. Preliminary data from the GTB-3550 (CD16/IL-15/CD33 TriKE) phase I/II trial (NCT03214666) in CD33^+^ AML/MDS demonstrated favorable safety with no severe CRS or neurotoxicity observed, along with evidence of NK cell expansion [39]. AFM28, a CD123/CD16A engager, also showed potent NK cell-mediated cytotoxicity against AML and high-risk MDS cells in preclinical studies [40]. Overall, NK cell engagers hold promise, but obstacles remain, particularly their short half-lives and susceptibility to CD16 shedding.

#### Adoptive NK cell transfer and CAR-NK cell therapy

In addition to T cells, adoptive NK cell transfer and chimeric antigen receptor-engineered NK cells (CAR-NK) provide an alternative approach, targeting AML-associated antigens such as CD33 and CD123 and are currently under evaluation in early-phase trials. NK cells do not require HLA matching and can be derived from NK-92 cell line, peripheral blood, induced pluripotent stem cells (iPSCs), or umbilical cord blood, allowing allogeneic application and cost-effective manufacturing for broader patient use (Fig. 2). The recent advances in clinical outcomes of adoptive NK cell transfer are summarized in the following reviews [41, 42]. Additionally, the incorporation of cytokines such as IL-15 and IL-21, which are critical for NK cell persistence, has been explored in CAR-NK cell therapies [43, 44]. Nevertheless, the systemic toxicity associated with soluble IL-15 secretion has been reported in mice [44], warranting the need for careful safety evaluation in future applications.

### Macrophages and myeloid-derived suppressor cells

CD47-SIRPa axis has been considered as an attractive targeting pathway, as CD47 expression is high in AML/MDS cells, which inhibits macrophages from clearance of malignant cells. However, a phase III randomized trial (ENHANCE-3 study, NCT05079230) targeting newly diagnosed elderly AML was recently reported to have failed to reach its efficacy endpoint and was early terminated [45]. This encourages researchers to focus on more other strategies to inhibit TAMs and MDSCs: (1) reducing the recruitment and infiltration, and (2) repolarization and functional remodeling (Fig. 2). In an AML mouse model, CCR2 antagonists markedly reduced M2-like macrophage infiltration in the spleen, indicating that blockade of the CCL2–CCR2 axis may represent a novel immunotherapeutic strategy [46]. CSF1R, which is highly expressed in TAMs, represents another promising therapeutic target. CSF1R signaling promotes paracrine secretion that sustains leukemic proliferation, and its inhibition significantly decreases both TAMs and AML cells [47].

## Conclusions and perspectives

The advancement of antitumor innate immunotherapy is essential for improving treatment outcomes in AML and MDS which are especially characterized by low mutational burdens and limited neoantigens. To develop more effective innate immunotherapies, it will be crucial to address two key aspects: the repression of tumor-associated immunosuppressive factors in AML/MDS cells and the enhancement of innate immune cell function. Recent studies have identified several immunosuppressive factors that hinder immune responses. For example, SETDB1 has been implicated as a NK cell suppressor in monocytic AML, while MED12, CCNC, and ARIH2 have been recognized as inhibitors of NK cell activity [13, 22]. Targeting these molecules represents a promising strategy for the development of novel therapeutic approaches.

Looking ahead, the integration of antitumor innate immunotherapy with molecular targeted therapies and T cell-based therapies is expected to pave the way for a new era in AML/MDS treatment. This combined approach has the potential to reduce reliance on conventional chemotherapy, offering a more precise and less toxic therapeutic option. Ultimately, such advancements may lead to the establishment of chemotherapy-free treatment regimens for AML and MDS, revolutionizing the standard of care for these challenging malignancies.
