# Correction: Recent advances of cell therapies for AML/MDS: exploring the therapeutic potential of innate immune cells in AML/MDS

**DOI:** 10.1007/s12185-025-04153-3

**Published:** 2026-01-09

**Authors:** Yu-Hsuan Chang, Susumu Goyama

**Affiliations:** Division of Molecular Oncology, Department of Computational Biology and Medical Sciences, Graduate School of Frontier Sciences, The University of Tokyo, 4-6-1 Shirokanedai, Minato-Ku, Tokyo, 108-8639 Japan

**Correction: International Journal of Hematology (2025)** https://doi.org/10.1007/s12185-025-04113-x

In Fig. 1 of this article “Immaute neutrophil” should have read “Immature neutrophil”. The corrected Fig. 1 is given in the correction.

**Figure:**
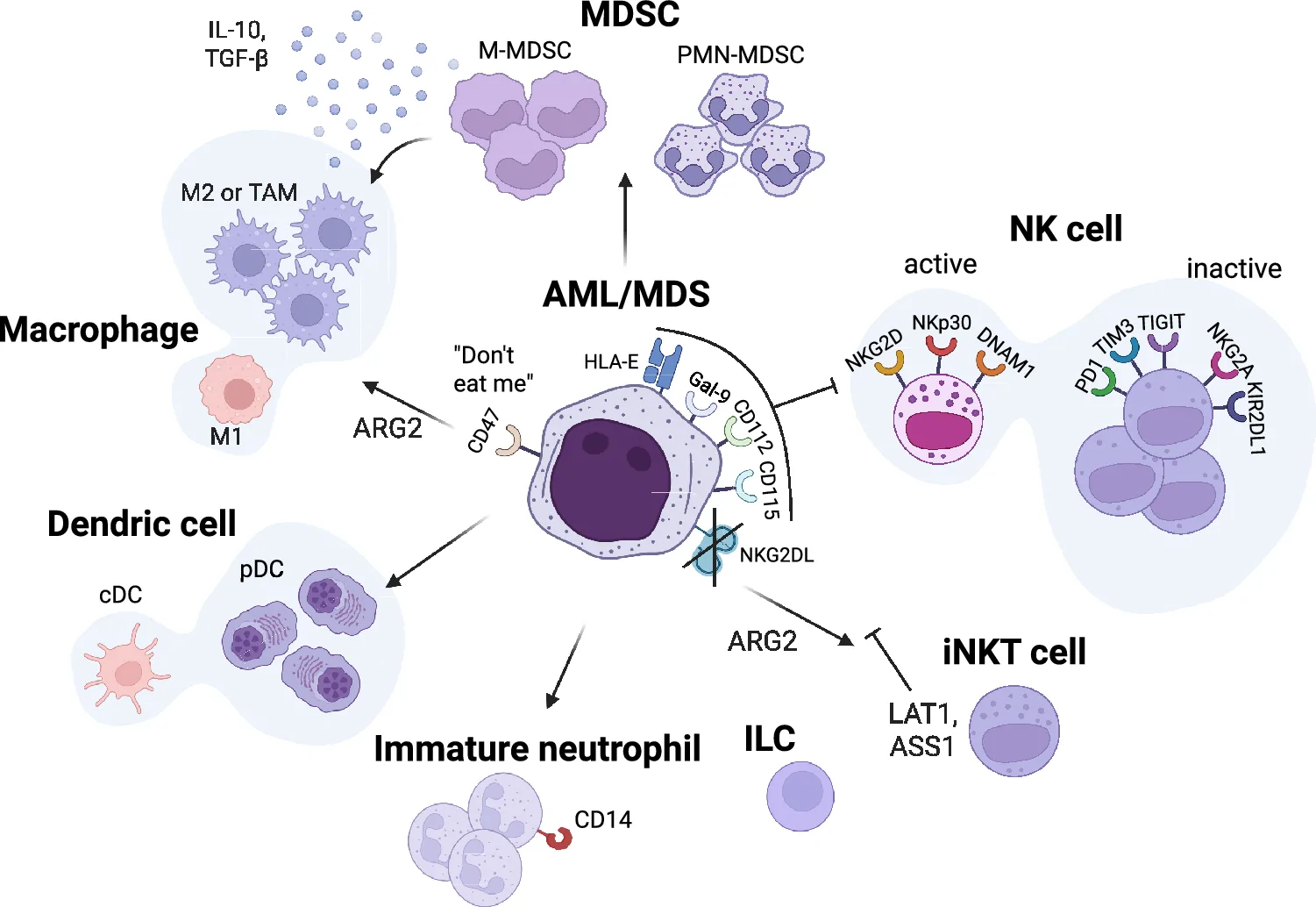

The original article has been corrected.

